# Chlorido­(4′-chloro-2,2′:6′,2′′-terpyridine-κ^3^ *N*,*N*′,*N*′′)(tri­fluoro­methane­sulfonato-κ*O*)zinc(II) aceto­nitrile monosolvate

**DOI:** 10.1107/S2414314620012924

**Published:** 2020-09-30

**Authors:** Rafael A. Adrian, Hadi D. Arman

**Affiliations:** a Department of Chemistry and Biochemistry, University of the Incarnate Word, San Antonio TX 78209, USA; b Department of Chemistry, The University of Texas at San Antonio, San Antonio TX 78249, USA; Katholieke Universiteit Leuven, Belgium

**Keywords:** crystal structure, zinc atom, chloro­terpyridine ligand, fivefold coordinated, tri­fluoro­methane­sulfonate salt

## Abstract

In the title complex, [Zn(CF_3_O_3_S)Cl(C_15_H_10_ClN_3_)]·CH_3_CN, the zinc(II) core is fivefold coordinated by one chloride, one tri­fluoro­methane­sulfonate O atom and three terpyridine N atoms in a slightly distorted square-pyramidal geometry. The structure provides a distinct example amongst other zinc(II) 4-chloro­terpyridine complexes because of the unusual planarity of the coordinated chloride, the short length of the Zn—N bond opposite to the chloride ligand [1.9572 (15) Å], and the presence of an elongated Zn—O bond [2.3911 (14) Å] in the coordinated tri­fluoro­methane­sulfonate ion. A molecule of acetonitrile is also found in the asymmetric unit of the title complex.

## Abstract 2

The title compound structure is a unique example of a zinc(II) metal center surrounded by a tridentate chloro­terpyridine ligand, a chloride and a coordinated tri­fluoro­methane­sulfonate in a distorted square-pyramidal geometry.

## Graphical abstract

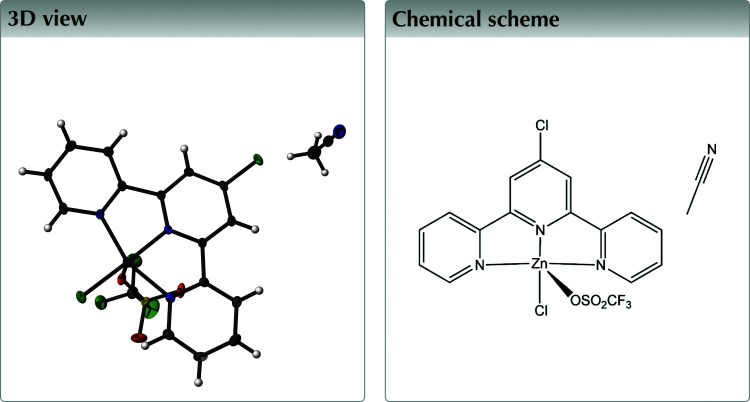

## Structure description

Substituted terpyridines such as 4′-chloro-2,2′:6′,2′′-terpyridine continue to be recognized as useful chelating ligands for many transition-metal ions, including platinum(II) (Qin *et al.*, 2019 ▸), copper(II) (Choroba *et al.*, 2019 ▸), cadmium(II) (Li *et al.*, 2020 ▸), and zinc(II) (Li *et al.*, 2019 ▸). Metal complexes containing zinc(II) and substituted terpyridines as chelating ligand have been shown to have promising anti­tumor activity (Liang *et al.*, 2019 ▸). Our research group inter­est currently lies in the synthesis of novel terpyridine–metal complexes with potential anti­tumor activity; as part of our research in this area, herein we describe the synthesis and structure of the title zinc(II) complex.

**Figure 1:**
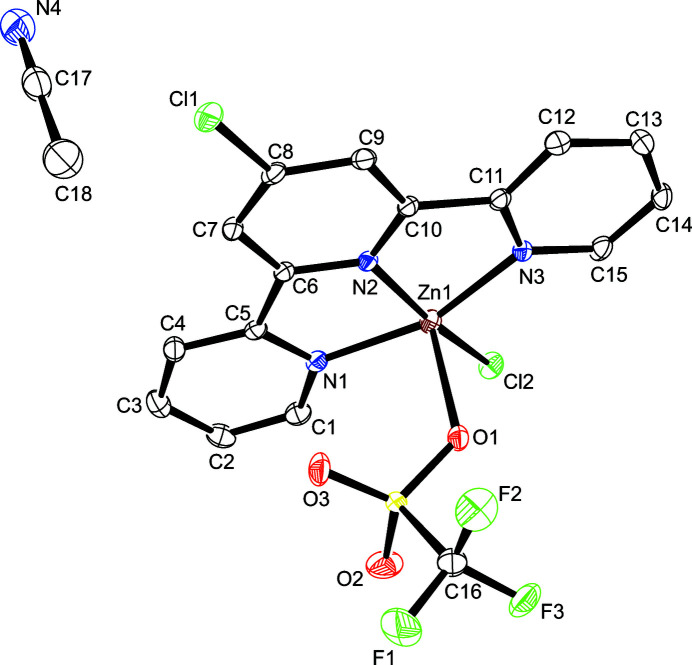
The mol­ecular structure of the title compound with displacement ellipsoids drawn at the 50% probability level; H atoms are omitted for clarity.

The asymmetric unit only contains the title compound, with four symmetry-related entities inside each unit cell. The zinc(II) ion shows a distorted square–pryramidal coordination environment defined by a tridentate 4-chloro­terpyridine ligand, a chloride, and an oxygen-coordinated tri­fluoro­methane­sulfonate (Fig. 1 ▸). The angle N2—Zn1—Cl2 of 168.69 (5)° is considerably closer to a planar geometry than the reported value (125.6°) in the only comparable zinc(II) 4-chloro­terpyridine structure currently available in the CSD (version 5.41 with update August 2020; Groom *et al.*, 2016 ▸; refcode HIVPOS; Huang & Qian, 2008 ▸). Another remarkable feature of the structure is that while Zn1—N3 and Zn1—N1 bond lengths [2.0403 (17) and 2.0468 (17) Å, respectively] are well within the values observed in others zinc(II) 4-chloro terpyridine complexes (Huang & Qian, 2008 ▸; Dutta *et al.*, 2019 ▸; You *et al.*, 2009 ▸), the Zn1—N2 bond length, across the chloride, is shorter [1.9572 (15) Å] and not comparable. The structure also features a coordinated tri­fluoro­methane­sulfonate anion that includes an elongated Zn—O bond of 2.3911 (14) Å (Gosiewska *et al.*, 2006 ▸). All relevant bonds and angles are presented in Table 1 ▸.

**Figure 2:**
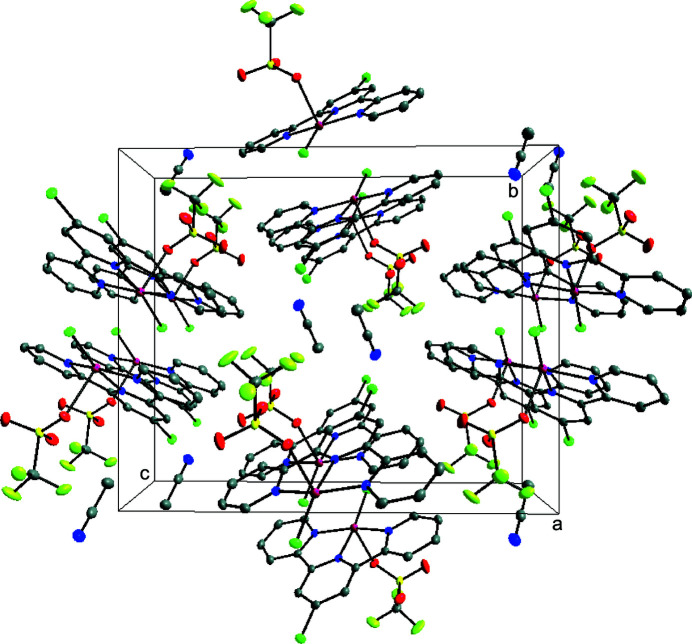
Perspective view of the packing structure of the title complex along the *a* axis.

The packing diagram reveals stacking of the asymmetric unit in columns along the *b* axis. These columns form an alternating pattern with the Cl1 atoms facing away from each other while the tri­fluoro­methane­sulfonate ions and aceto­nitrile mol­ecules occupying the space between the stacked zinc(II) 4-chloro­terpyridine units. Adjacent columns also alternate directions in the crystal lattice (Fig. 2 ▸).

## Synthesis and crystallization

4′-Chloro-2,2′:6′,2′′-terpyridine (0.200 g, 0.747 mmol) was suspended in 30 ml of aceto­nitrile and stirred for 10 min. ZnCl_2_ (0.102 g, 0.747 mmol) was added to the suspension and heated under stirring at 323 K for 1 h. AgOTf (0.384 g, 1.49 mmol) was added to the mixture and stirred without heating for 30 min. After the removal of AgCl by filtration using a 0.45 µm PTFE syringe filter, the resulting clear solution was used to grow crystals by vapor diffusion with diethyl ether at 278 K.

## Refinement

Crystal data, data collection and structure refinement details are summarized in Table 2 ▸.

## Supplementary Material

Crystal structure: contains datablock(s) I. DOI: 10.1107/S2414314620012924/vm4046sup1.cif

Structure factors: contains datablock(s) I. DOI: 10.1107/S2414314620012924/vm4046Isup2.hkl

Supporting information file. DOI: 10.1107/S2414314620012924/vm4046Isup3.mol

3D View of title complex. DOI: 10.1107/S2414314620012924/vm4046sup4.ps

CCDC reference: 2033215

Additional supporting information:  crystallographic information; 3D view; checkCIF report

## Figures and Tables

**Table 1:** Selected geometric parameters (Å, °)

Zn1—Cl2	2.2206 (6)	Zn1—N2	1.9572 (15)
Zn1—O1	2.3911 (14)	Zn1—N1	2.0468 (17)
Zn1—N3	2.0403 (17)		
			
Cl2—Zn1—O1	98.13 (4)	N1—Zn1—Cl2	99.19 (5)
N2—Zn1—Cl2	168.69 (5)	N1—Zn1—O1	92.17 (6)
N2—Zn1—O1	93.18 (6)	N3—Zn1—Cl2	99.67 (5)
N2—Zn1—N1	79.97 (7)	N3—Zn1—O1	93.68 (6)
N2—Zn1—N3	79.84 (6)	N3—Zn1—N1	159.24 (6)

**Table 2:** Experimental details

Crystal data	
Chemical formula	[Zn(CF_3_O_3_S)Cl(C_15_H_10_ClN_3_)]·C_2_H_3_N
*M*_r_	558.65
Crystal system, space group	Monoclinic, *P*2_1_/*n*
Temperature (K)	98
*a*, *b*, *c* (Å)	7.6550 (14), 15.329 (3), 18.486 (4)
β (°)	92.088 (7)
*V* (Å^3^)	2167.8 (8)
*Z*	4
Radiation type	Mo *K*α
μ (mm^−1^)	1.53
Crystal size (mm)	0.5 × 0.23 × 0.13

Data collection	
Diffractometer	Rigaku Saturn724
Absorption correction	Multi-scan (*ABSCOR*; Higashi, 1995 ▸)
*T*_min_, *T*_max_	0.287, 1.000
No. of measured, independent and observed [*I* > 2σ(*I*)] reflections	15157, 4424, 4053
*R*_int_	0.028
(sin θ/λ)_max_ (Å^−1^)	0.625

Refinement	
*R*[*F* ^2^ > 2σ(*F* ^2^)], *wR*(*F* ^2^), *S*	0.027, 0.075, 1.05
No. of reflections	4424
No. of parameters	290
H-atom treatment	H-atom parameters constrained
Δρ_max_, Δρ_min_ (e Å^−3^)	0.48, −0.46

Computer programs: *CrysAlis PRO* (Rigaku OD, 2015 ▸), *OLEX2* (Dolomanov *et al.*, 2009 ▸) and *SHELXL* (Sheldrick, 2015 ▸).

## full crystallographic data

### Crystal data

**Table:**

[Zn(CF_3_O_3_S)Cl(C_15_H_10_ClN_3_)]·C_2_H_3_N	*F*(000) = 1120
*M**_r_* = 558.65	*D*_x_ = 1.712 Mg m^−^^3^
Monoclinic, *P*2_1_/*n*	Mo *K*α radiation, λ = 0.71075 Å
*a* = 7.6550 (14) Å	Cell parameters from 10648 reflections
*b* = 15.329 (3) Å	θ = 3.0–27.4°
*c* = 18.486 (4) Å	µ = 1.53 mm^−^^1^
β = 92.088 (7)°	*T* = 98 K
*V* = 2167.8 (8) Å^3^	Chunk, blue
*Z* = 4	0.5 × 0.23 × 0.13 mm

### Data collection

**Table:**

Rigaku Saturn724 diffractometer	4424 independent reflections
Radiation source: Sealed Tube	4053 reflections with *I* > 2σ(*I*)
Graphite Monochromator monochromator	*R*_int_ = 0.028
Detector resolution: 28.5714 pixels mm^-1^	θ_max_ = 26.4°, θ_min_ = 3.0°
profile data from ω–scans	*h* = −9→9
Absorption correction: multi-scan (ABSCOR; Higashi, 1995)	*k* = −19→19
*T*_min_ = 0.287, *T*_max_ = 1.000	*l* = −23→22
15157 measured reflections	

### Refinement

**Table:**

Refinement on *F*^2^	0 restraints
Least-squares matrix: full	Hydrogen site location: inferred from neighbouring sites
*R*[*F*^2^ > 2σ(*F*^2^)] = 0.027	H-atom parameters constrained
*wR*(*F*^2^) = 0.075	*w* = 1/[σ^2^(*F*_o_^2^) + (0.040*P*)^2^ + 1.6585*P*] where *P* = (*F*_o_^2^ + 2*F*_c_^2^)/3
*S* = 1.05	(Δ/σ)_max_ = 0.001
4424 reflections	Δρ_max_ = 0.48 e Å^−^^3^
290 parameters	Δρ_min_ = −0.46 e Å^−^^3^

### Special details

**Table:**

Geometry. All esds (except the esd in the dihedral angle between two l.s. planes) are estimated using the full covariance matrix. The cell esds are taken into account individually in the estimation of esds in distances, angles and torsion angles; correlations between esds in cell parameters are only used when they are defined by crystal symmetry. An approximate (isotropic) treatment of cell esds is used for estimating esds involving l.s. planes.

### Fractional atomic coordinates and isotropic or equivalent isotropic displacement parameters (Å^2^)

**Table:**

	*x*	*y*	*z*	*U*_iso_*/*U*_eq_	
Zn1	0.57879 (3)	0.10461 (2)	0.54368 (2)	0.01503 (8)	
Cl2	0.77504 (6)	0.00527 (3)	0.57903 (3)	0.01862 (11)	
S1	0.66102 (6)	0.27610 (3)	0.66109 (3)	0.01438 (11)	
Cl1	−0.06371 (6)	0.33529 (3)	0.41880 (3)	0.02027 (12)	
F3	0.96127 (16)	0.36209 (9)	0.66388 (8)	0.0307 (3)	
F2	0.75796 (19)	0.41967 (9)	0.59444 (8)	0.0354 (3)	
O1	0.71851 (17)	0.22976 (9)	0.59695 (8)	0.0176 (3)	
F1	0.7465 (2)	0.43216 (10)	0.71074 (9)	0.0462 (4)	
O3	0.48031 (18)	0.30461 (10)	0.65635 (9)	0.0249 (3)	
N2	0.38097 (19)	0.17251 (10)	0.50413 (9)	0.0119 (3)	
N1	0.4111 (2)	0.09391 (10)	0.62698 (9)	0.0137 (3)	
N3	0.66449 (19)	0.13089 (10)	0.44275 (9)	0.0132 (3)	
O2	0.7203 (2)	0.23764 (11)	0.72904 (9)	0.0312 (4)	
C6	0.2382 (2)	0.18139 (12)	0.54416 (10)	0.0127 (4)	
C11	0.5531 (2)	0.18136 (12)	0.40107 (11)	0.0135 (4)	
C10	0.3898 (2)	0.20620 (12)	0.43760 (10)	0.0125 (4)	
N4	−0.4601 (3)	0.42556 (13)	0.40405 (11)	0.0290 (4)	
C5	0.2542 (2)	0.13511 (12)	0.61493 (11)	0.0129 (4)	
C7	0.0949 (2)	0.23059 (12)	0.51838 (11)	0.0147 (4)	
H7	−0.0047	0.2369	0.5452	0.018*	
C15	0.8157 (2)	0.10331 (12)	0.41444 (11)	0.0163 (4)	
H15	0.8913	0.0686	0.4425	0.020*	
C8	0.1082 (2)	0.26963 (12)	0.45068 (11)	0.0144 (4)	
C1	0.4419 (2)	0.05299 (12)	0.69072 (11)	0.0177 (4)	
H1	0.5490	0.0256	0.6994	0.021*	
C4	0.1227 (2)	0.13329 (12)	0.66510 (11)	0.0167 (4)	
H4	0.0155	0.1601	0.6552	0.020*	
C9	0.2528 (2)	0.25749 (12)	0.40792 (10)	0.0143 (4)	
H9	0.2582	0.2821	0.3621	0.017*	
C12	0.5901 (2)	0.20541 (13)	0.33050 (11)	0.0170 (4)	
H12	0.5120	0.2394	0.3030	0.020*	
C14	0.8612 (2)	0.12554 (13)	0.34456 (11)	0.0183 (4)	
H14	0.9661	0.1062	0.3264	0.022*	
C3	0.1562 (3)	0.09007 (13)	0.73072 (12)	0.0204 (4)	
H3	0.0710	0.0878	0.7653	0.024*	
C2	0.3178 (3)	0.05051 (13)	0.74399 (11)	0.0204 (4)	
H2	0.3427	0.0227	0.7879	0.024*	
C17	−0.3767 (3)	0.48293 (14)	0.42493 (12)	0.0227 (4)	
C13	0.7479 (3)	0.17715 (13)	0.30196 (11)	0.0191 (4)	
H13	0.7765	0.1926	0.2552	0.023*	
C16	0.7872 (3)	0.37808 (14)	0.65711 (12)	0.0224 (4)	
C18	−0.2688 (3)	0.55655 (15)	0.45103 (14)	0.0304 (5)	
H18A	−0.2941	0.6070	0.4217	0.046*	
H18B	−0.1474	0.5418	0.4480	0.046*	
H18C	−0.2944	0.5690	0.5004	0.046*	

### Atomic displacement parameters (Å^2^)

**Table:**

	*U*^11^	*U*^22^	*U*^33^	*U*^12^	*U*^13^	*U*^23^
Zn1	0.01194 (12)	0.01654 (12)	0.01657 (13)	0.00242 (8)	−0.00006 (9)	−0.00003 (9)
Cl2	0.0152 (2)	0.0169 (2)	0.0236 (3)	0.00591 (16)	−0.00090 (18)	0.00110 (18)
S1	0.0125 (2)	0.0189 (2)	0.0118 (2)	−0.00271 (16)	0.00134 (17)	−0.00214 (18)
Cl1	0.0152 (2)	0.0225 (2)	0.0228 (3)	0.00850 (17)	−0.00349 (18)	0.00070 (19)
F3	0.0176 (6)	0.0366 (7)	0.0377 (8)	−0.0105 (5)	−0.0020 (5)	−0.0001 (6)
F2	0.0393 (8)	0.0246 (6)	0.0420 (9)	−0.0027 (6)	−0.0007 (7)	0.0113 (6)
O1	0.0132 (6)	0.0204 (7)	0.0196 (7)	−0.0014 (5)	0.0044 (5)	−0.0056 (6)
F1	0.0517 (9)	0.0371 (8)	0.0507 (10)	−0.0152 (7)	0.0157 (8)	−0.0296 (8)
O3	0.0139 (7)	0.0297 (8)	0.0315 (9)	0.0014 (6)	0.0075 (6)	−0.0083 (7)
N2	0.0093 (7)	0.0134 (7)	0.0128 (8)	0.0009 (5)	−0.0005 (6)	−0.0020 (6)
N1	0.0120 (7)	0.0142 (7)	0.0149 (8)	−0.0011 (6)	−0.0003 (6)	−0.0004 (6)
N3	0.0105 (7)	0.0160 (7)	0.0131 (8)	0.0005 (6)	0.0006 (6)	−0.0029 (6)
O2	0.0346 (9)	0.0410 (9)	0.0174 (8)	−0.0117 (7)	−0.0057 (7)	0.0080 (7)
C6	0.0100 (8)	0.0147 (8)	0.0135 (9)	−0.0009 (7)	−0.0003 (7)	−0.0031 (7)
C11	0.0107 (8)	0.0143 (8)	0.0155 (9)	−0.0009 (7)	−0.0004 (7)	−0.0036 (7)
C10	0.0104 (8)	0.0137 (8)	0.0133 (9)	−0.0005 (7)	−0.0005 (7)	−0.0022 (7)
N4	0.0269 (10)	0.0310 (10)	0.0293 (11)	0.0007 (8)	0.0027 (8)	−0.0062 (9)
C5	0.0122 (8)	0.0123 (8)	0.0141 (9)	−0.0018 (7)	−0.0015 (7)	−0.0015 (7)
C7	0.0102 (8)	0.0176 (9)	0.0164 (10)	0.0006 (7)	0.0010 (7)	−0.0039 (8)
C15	0.0111 (8)	0.0166 (9)	0.0212 (10)	0.0019 (7)	−0.0004 (7)	−0.0041 (8)
C8	0.0104 (8)	0.0147 (8)	0.0177 (10)	0.0021 (7)	−0.0035 (7)	−0.0028 (7)
C1	0.0173 (9)	0.0169 (9)	0.0186 (10)	0.0015 (7)	−0.0027 (8)	0.0014 (8)
C4	0.0142 (9)	0.0177 (9)	0.0182 (10)	−0.0001 (7)	0.0030 (7)	−0.0019 (8)
C9	0.0139 (8)	0.0160 (9)	0.0127 (9)	−0.0004 (7)	−0.0022 (7)	−0.0006 (7)
C12	0.0155 (9)	0.0198 (9)	0.0156 (10)	0.0006 (7)	−0.0011 (7)	−0.0003 (8)
C14	0.0123 (9)	0.0222 (10)	0.0207 (11)	0.0011 (7)	0.0047 (8)	−0.0065 (8)
C3	0.0232 (10)	0.0200 (9)	0.0183 (11)	−0.0015 (8)	0.0066 (8)	−0.0013 (8)
C2	0.0275 (10)	0.0200 (9)	0.0134 (10)	0.0001 (8)	−0.0011 (8)	0.0031 (8)
C17	0.0219 (10)	0.0262 (11)	0.0203 (11)	0.0057 (9)	0.0044 (8)	0.0005 (9)
C13	0.0181 (9)	0.0247 (10)	0.0147 (10)	−0.0025 (8)	0.0037 (8)	−0.0033 (8)
C16	0.0220 (10)	0.0210 (10)	0.0242 (12)	−0.0037 (8)	0.0023 (9)	−0.0062 (9)
C18	0.0314 (12)	0.0291 (12)	0.0309 (13)	−0.0062 (9)	0.0034 (10)	−0.0023 (10)

### Geometric parameters (Å, º)

**Table:**

Zn1—Cl2	2.2206 (6)	N4—C17	1.145 (3)
Zn1—O1	2.3911 (14)	C5—C4	1.394 (3)
Zn1—N3	2.0403 (17)	C7—H7	0.9300
Zn1—N2	1.9572 (15)	C7—C8	1.394 (3)
Zn1—N1	2.0468 (17)	C15—H15	0.9300
S1—O1	1.4636 (14)	C15—C14	1.393 (3)
S1—O3	1.4503 (15)	C8—C9	1.396 (3)
S1—O2	1.4455 (16)	C1—H1	0.9300
S1—C16	1.840 (2)	C1—C2	1.394 (3)
Cl1—C8	1.7420 (18)	C4—H4	0.9300
F3—C16	1.357 (2)	C4—C3	1.398 (3)
F2—C16	1.334 (3)	C9—H9	0.9300
F1—C16	1.338 (3)	C12—H12	0.9300
N2—C6	1.349 (2)	C12—C13	1.404 (3)
N2—C10	1.338 (2)	C14—H14	0.9300
N1—C5	1.368 (2)	C14—C13	1.396 (3)
N1—C1	1.348 (3)	C3—H3	0.9300
N3—C11	1.368 (2)	C3—C2	1.391 (3)
N3—C15	1.356 (2)	C2—H2	0.9300
C6—C5	1.489 (3)	C17—C18	1.470 (3)
C6—C7	1.400 (3)	C13—H13	0.9300
C11—C10	1.491 (3)	C18—H18A	0.9600
C11—C12	1.395 (3)	C18—H18B	0.9600
C10—C9	1.406 (3)	C18—H18C	0.9600
			
Cl2—Zn1—O1	98.13 (4)	N3—C15—C14	121.86 (18)
N2—Zn1—Cl2	168.69 (5)	C14—C15—H15	119.1
N2—Zn1—O1	93.18 (6)	C7—C8—Cl1	118.23 (14)
N2—Zn1—N1	79.97 (7)	C7—C8—C9	122.42 (17)
N2—Zn1—N3	79.84 (6)	C9—C8—Cl1	119.36 (15)
N1—Zn1—Cl2	99.19 (5)	N1—C1—H1	119.0
N1—Zn1—O1	92.17 (6)	N1—C1—C2	121.91 (18)
N3—Zn1—Cl2	99.67 (5)	C2—C1—H1	119.0
N3—Zn1—O1	93.68 (6)	C5—C4—H4	120.9
N3—Zn1—N1	159.24 (6)	C5—C4—C3	118.23 (18)
O1—S1—C16	101.90 (9)	C3—C4—H4	120.9
O3—S1—O1	114.30 (9)	C10—C9—H9	121.7
O3—S1—C16	104.02 (10)	C8—C9—C10	116.70 (18)
O2—S1—O1	114.32 (10)	C8—C9—H9	121.7
O2—S1—O3	116.35 (10)	C11—C12—H12	120.8
O2—S1—C16	103.45 (10)	C11—C12—C13	118.47 (18)
S1—O1—Zn1	125.44 (8)	C13—C12—H12	120.8
C6—N2—Zn1	118.69 (13)	C15—C14—H14	120.3
C10—N2—Zn1	118.96 (12)	C15—C14—C13	119.38 (18)
C10—N2—C6	122.32 (16)	C13—C14—H14	120.3
C5—N1—Zn1	114.29 (13)	C4—C3—H3	120.2
C1—N1—Zn1	126.91 (13)	C2—C3—C4	119.56 (19)
C1—N1—C5	118.78 (17)	C2—C3—H3	120.2
C11—N3—Zn1	114.34 (12)	C1—C2—H2	120.4
C15—N3—Zn1	126.80 (13)	C3—C2—C1	119.22 (19)
C15—N3—C11	118.86 (17)	C3—C2—H2	120.4
N2—C6—C5	113.06 (16)	N4—C17—C18	179.4 (3)
N2—C6—C7	120.53 (18)	C12—C13—H13	120.4
C7—C6—C5	126.41 (17)	C14—C13—C12	119.24 (19)
N3—C11—C10	113.87 (17)	C14—C13—H13	120.4
N3—C11—C12	122.19 (17)	F3—C16—S1	110.98 (14)
C12—C11—C10	123.93 (17)	F2—C16—S1	111.67 (14)
N2—C10—C11	112.90 (16)	F2—C16—F3	107.43 (17)
N2—C10—C9	120.82 (17)	F2—C16—F1	108.14 (18)
C9—C10—C11	126.26 (18)	F1—C16—S1	111.11 (15)
N1—C5—C6	113.98 (16)	F1—C16—F3	107.33 (17)
N1—C5—C4	122.25 (18)	C17—C18—H18A	109.5
C4—C5—C6	123.78 (17)	C17—C18—H18B	109.5
C6—C7—H7	121.5	C17—C18—H18C	109.5
C8—C7—C6	117.06 (17)	H18A—C18—H18B	109.5
C8—C7—H7	121.5	H18A—C18—H18C	109.5
N3—C15—H15	119.1	H18B—C18—H18C	109.5
			
Zn1—N2—C6—C5	−0.7 (2)	O2—S1—C16—F3	55.57 (17)
Zn1—N2—C6—C7	178.98 (13)	O2—S1—C16—F2	175.40 (15)
Zn1—N2—C10—C11	3.2 (2)	O2—S1—C16—F1	−63.78 (18)
Zn1—N2—C10—C9	−178.17 (13)	C6—N2—C10—C11	−174.71 (16)
Zn1—N1—C5—C6	−1.36 (19)	C6—N2—C10—C9	3.9 (3)
Zn1—N1—C5—C4	178.72 (14)	C6—C5—C4—C3	−177.78 (17)
Zn1—N1—C1—C2	179.44 (14)	C6—C7—C8—Cl1	−177.17 (13)
Zn1—N3—C11—C10	−0.70 (19)	C6—C7—C8—C9	3.2 (3)
Zn1—N3—C11—C12	−179.68 (14)	C11—N3—C15—C14	0.6 (3)
Zn1—N3—C15—C14	−179.87 (14)	C11—C10—C9—C8	177.31 (17)
Cl1—C8—C9—C10	177.93 (13)	C11—C12—C13—C14	0.6 (3)
O1—S1—C16—F3	−63.33 (16)	C10—N2—C6—C5	177.25 (16)
O1—S1—C16—F2	56.51 (16)	C10—N2—C6—C7	−3.1 (3)
O1—S1—C16—F1	177.33 (16)	C10—C11—C12—C13	−179.40 (17)
O3—S1—O1—Zn1	−54.32 (13)	C5—N1—C1—C2	0.9 (3)
O3—S1—C16—F3	177.58 (14)	C5—C6—C7—C8	179.16 (17)
O3—S1—C16—F2	−62.58 (17)	C5—C4—C3—C2	0.0 (3)
O3—S1—C16—F1	58.24 (18)	C7—C6—C5—N1	−178.30 (17)
N2—C6—C5—N1	1.3 (2)	C7—C6—C5—C4	1.6 (3)
N2—C6—C5—C4	−178.75 (17)	C7—C8—C9—C10	−2.4 (3)
N2—C6—C7—C8	−0.4 (3)	C15—N3—C11—C10	178.92 (15)
N2—C10—C9—C8	−1.1 (3)	C15—N3—C11—C12	−0.1 (3)
N1—C5—C4—C3	2.1 (3)	C15—C14—C13—C12	−0.1 (3)
N1—C1—C2—C3	1.1 (3)	C1—N1—C5—C6	177.34 (16)
N3—C11—C10—N2	−1.5 (2)	C1—N1—C5—C4	−2.6 (3)
N3—C11—C10—C9	179.96 (17)	C4—C3—C2—C1	−1.5 (3)
N3—C11—C12—C13	−0.5 (3)	C12—C11—C10—N2	177.45 (17)
N3—C15—C14—C13	−0.5 (3)	C12—C11—C10—C9	−1.1 (3)
O2—S1—O1—Zn1	83.30 (11)	C16—S1—O1—Zn1	−165.84 (10)
